# T Cell IFN-γ Suppression Following Alcohol and Burn Injury Is Independent of miRNA155

**DOI:** 10.1371/journal.pone.0105314

**Published:** 2014-08-15

**Authors:** Xiaoling Li, Juan L. Rendon, Mashkoor A. Choudhry

**Affiliations:** 1 Alcohol Research Program, Loyola University Chicago Health Sciences Division, Maywood, Illinois, United States of America; 2 Burn & Shock Trauma Research Institute, Loyola University Chicago Health Sciences Division, Maywood, Illinois, United States of America; 3 Department of Surgery, Loyola University Chicago Health Sciences Division, Maywood, Illinois, United States of America; 4 Deparmtent of Microbiology and Immunology, Loyola University Chicago Health Sciences Division, Maywood, Illinois, United States of America; Georgia Regents University, United States of America

## Abstract

miRNA155 has been implicated in normal T cell function and their differentiations into the Th1 subtype. We have shown that acute alcohol (ethanol) intoxication combined with burn injury suppresses T cell IFN-γ release. Herein, we examined whether the decrease in IFN-γ is resulted from altered expression of miRNA155 and transcription factors -NFAT, Tbx21, Jun and Fos - in T cells following ethanol and burn injury. Mice received ethanol (∼3 g/Kg) 4 hours prior to ∼12.5% total body surface area sham or burn injury and were sacrificed one day after injury. Splenic T cells were harvested and cultured with anti-CD3 (2 µg/ml) in the presence or absence of rIL-12 (10 ng/ml) or PMA (10 ng/ml) plus ionomycin (50 ng/ml) for 48 hours. We observed a significant decrease in miRNA155, NFAT, Tbx21, Jun and Fos expression as well as IFN-γ release in T cells cultured with anti-CD3 following ethanol and burn injury compared with shams. The co-treatment of T cells with rIL-12 prevented the decrease in IFN-γ and NFAT, Tbx21, Jun and Fos, but not miRNA155. In contrast, the co-treatment with PMA plus ionomycin normalized the expression of NFAT. It did not prevent the decrease in IFN-γ, Tbx21, Jun, Fos and miRNA155. Finally, results obtained in miRNA155^-/-^ mice did not show any change in T cell release of IFN-γ or expression of nuclear factors compared to wildtype mice. Together, these findings suggest that while ethanol and burn injury decreases the expression of miRNA155, it may not be involved in decreased IFN-γ under those conditions.

## Introduction

Worldwide, alcohol abuse is a major social and health problem. Alcohol abuse, particularly chronic alcohol consumption, impairs immune cell function, including T cells, macrophages, dendritic cells (DCs), B cells and neutrophils [1]–[5]. Acute alcohol intoxication is associated with about 50% of the nearly one million burn injury cases reported annually in the United States [1], [2], [6]–[8]. These studies further suggest that these patients are more susceptible to infection, require more surgical procedures, have longer hospital stays, and exhibit higher mortality as compared to burn patients who sustained a similar extent of injury without alcohol consumption [1], [2], [6]–[8]. Previous studies from our laboratory have shown that acute alcohol (ethanol) intoxication combined with burn injury suppresses T cell proliferation, IL-2, IFN-γ, IL-17 and IL-22 production in cells isolated from mesenteric lymph nodes (MLN), Peyer's patches (PP) and spleens [9]–[14]. This was accompanied with increased gut leakiness and bacterial translocation [9], [10], [15], [16], which further confound the pathogenesis associated with burn injury. We further demonstrated that treatment of T cells with recombinant IL-12 (rIL-12) prevented the decrease in IFN-γ following ethanol intoxication and burn injury [12]. However, the mechanism underlying T cell suppression after ethanol and burn injury remains unclear.

The process of T cell activation, proliferation, and further differentiation into various subsets is complex and mediated by multiple layers of signaling pathways [2], [17]–[19]. The T cell receptor (TCR) associates with the CD3 molecule, which primarily recognizes antigens presented in context of major histocompatibility complex (MHC) molecules expressed on antigen-presenting cells (APCs). This interaction results in phosphorylation of TCR-associated protein tyrosine kinases (PTK), including P56lck and p59fyn, as well as 70-kd zeta-associated protein kinase (Zap-70). This further leads to the phosphorylation of phospholipase C-γ (PLC-γ). PLC-γ hydrolyzes phosphatidylinositol 4,5-bisphosphate (PIP2) into inositol 1,4,5-trisphosphate (IP3) and 1,2-diacylglycerol (DAG), which subsequently activates the downstream MAP kinase pathways, namely p38, extracellular signal-regulated protein kinase (ERK) and c-Jun N-terminal kinase (JNK) [2], [17], [18]. These pathways activate downstream transcription factors, including NFAT, AP-1 T-bet, and Tbx21, which ultimately induce T cell proliferation, activation and further differentiation into various T cell subsets by cytokine production [2], [17]–[21]. We have shown a role of MAPK in suppressed T cell IFN-γ release after alcohol and burn injury [11], [12].

Recent findings suggest that T cell activation and differentiation into various subsets is further controlled by a class of small non-coding RNAs referred to as microRNAs (miRNAs) [22]–[25]. mRNAs are small (∼20–25 nucleotides), single-stranded noncoding RNAs. They bind to the 3′ untranslated regions of specific target mRNAs to regulate gene expression at the posttranscriptional level, and affect many biological processes including innate and adaptive immune cell development and function [25]–[27]. Each miRNA can bind multiple target mRNAs to mediate gene expression and function. Several miRNAs (e.g. miR126, miR155, mir181a, miR182 etc.) are identified in T cells and are shown to regulate various aspects of T cell development and differentiation. Studies have shown that miRNA155 is required for normal T cell function and differentiation into Th1, Th2 and Th17 [22]–[25]. miRNA155 upregulates IFN-γ production in NK cells stimulated with IL-12 and IL-18 [28]. T cells in miRNA155^-/-^ mice are biased toward Th2 differentiation, which suggests that miRNA155 promotes differentiation of T cells into Th1 cells [23], [25]. miRNA155 is also regulated by antigens, cytokines, hormones and bacterial production [29]–[31]. In this study, we determined whether acute ethanol combined with burn injury alters miRNA155 expression and the transcription factors NFAT, Tbx21, Jun and Fos involved in T cell activation and IFN-γ release. IL-12 is an important cytokine involved in Th1 differentiation and IFN-γ production [32], [33]. Utilizing ex vivo approaches, we examined whether treatment of T cells with rIL-12 influences the expression of transcription. We also treated T cells with PMA combined with ionomycin to determine whether direct activation of PKC and calcium signaling dependent pathways modulate the expression of miRNA155 and nuclear factors following ethanol combined with burn injury. Finally, we used miRNA155^-/-^ mice to confirm the role of miRNA155 in T cell release of IFN-γ.

## Materials and Methods

### Animals and Reagents

Male C57/BL6 mice (22–25 g) were obtained from Harlan Laboratories (Indianapolis, IN). miRNA155 knockout mice (Mir155tm1.1Rsky/J) and C57/BL6 wild-type mice were obtained from Jackson Laboratories (Bar Harbor, ME). IFN-γ ELISA kits were obtained from BD Biosciences (San Diego, CA). Primers to Nfatc 2 (NFAT), Tbx 21, Jun, Fos, 18 s, GAPDH, miRNA155, and U6 snRNA were obtained from Life Technologies (Grand Island, NY). MirVana miRNA Isolation Kit, TaqMan MicroRNA Assays, TaqMan MicroRNA Reverse Transcription Kit, High Capacity cDNA Reverse Transcription Kit, TaqMan Gene Expression Master Mix and TaqMan Universal Master Mix II were obtained from Life Technologies.

### Mouse Model of Acute Ethanol Intoxication and Burn Injury

As described previously [13], [34], 22–25 g male mice were randomly divided into four groups: sham vehicle, sham ethanol, burn vehicle and burn ethanol. Mice were gavaged orally with ethanol (∼3 g/Kg) or water (vehicle). At four hours after the gavage, a time when blood ethanol levels are in the range of 90–100 mg/dL, mice were anesthetized with a mixture of ketamine (80 mg/Kg) and xylazine (1.25 mg/Kg) by intraperitoneal injection (IP) and transferred into a template fabricated to expose ∼12.5% of the total body surface area (TBSA). For burn injury, mice were immersed in ∼90°C water bath for ∼7 seconds. For sham injury, mice were immersed in lukewarm water. All mice were dried immediately and resuscitated with 1.0 ml physiological saline by IP injection. After recovery from anesthesia, the mice were returned to their cages and allowed food and water *ad libitum*. All the animal procedures were carried out in adherence to the National Institutes of Health 2011 *Guide for the Care and Use of Laboratory Animals* and were approved by the Loyola University Chicago Health Sciences Division Institutional Animal Care and Use Committee.

### T Cell Isolation

One day after injury, mice were sacrificed and spleens were collected. To prepare single cell suspensions, spleens were gently crushed in HBSS solution (Fisher Scientific, Pittsburgh, PA) supplemented with 10 mMol/L HEPES, 50 µg/ml gentamicin and 100 U/ml penicillin with 100 µg/ml streptomycin [12], [13]. Cell suspensions were centrifuged at 290 *g* for 15 min at 10°C. Supernatants were discarded and cells were reconstituted in 9 ml of sterile-distilled H_2_O following by 1 ml of 10× phosphate-buffered saline (PBS) to lyse red blood cells. 10^6^–10^7^ total cells were resuspended in 90 µl of staining buffer (PBS containing 0.5% BSA and 2 mMol/L EDTA) and incubated with 10 µl of CD90 (Thy1.2) MicroBeads (MiltenyiBiotec, Auburn, CA) for 15 min at 4°C. The cells were washed with staining buffer and run through separation columns (Miltenyi Biotec) in a magnetic field. Purified T cells were obtained by flushing out magnetically labeled cells from the separation columns [13].

### Determination of T Cell IFN-γ Levels

As previously described [11]–[13], MicroBead purified T cells (5×10^5^ cells/well) were cultured in RPMI-1640 supplemented with 2 mMol/L L-glutamine, 10 mMol/L HEPES, 50 µg/ml gentamicin, 100 U/ml penicillin with 100 µg/ml streptomycin and 10% FCS in 96-well plates in the presence of plate-bound anti-CD3 (2 µg/ml) with/without PMA (10 ng/ml) plus ionomycin (50 ng/ml) or rIL-12 (10 ng/ml) at 37°C and 5% CO_2_ for 48 h. Following culture, supernatants were harvested to test IFN-γ levels by using ELISA kits according to the manufacturer's instructions. T cells were collected for analyses of transcription factors and miRNA155.

### Determination of T Cell Transcription Factor mRNA Expression

T cell large RNA and enriched small RNA were isolated separately by using mirVana miRNA Isolation Kit according to the manufacturer's instructions. The RNA concentration was determined by using a Nanodrop spectrophotometer (Thermo Scientific) and A260/A280 ratio was 1.7–2.0. For transcription factor analysis, 0.5–1 µg of the large RNA was used for cDNA reverse transcription by using a High Capacity cDNA Reverse Transcription Kit according to the manufacturer's instructions. The expression of transcription factors (NFAT, Tbx21, Jun and Fos) was analyzed by RT-PCR with specific primers to mouse Nfatc2 (NFAT), Tbx21, Jun and Fos. The 18 s or GAPDH were used as the endogenous control. All the samples were amplified at 50°C for 2 min and 95°C for 10 min followed by 40 cycles at 95°C 15 sec and 60°C 1 min and were performed by using 7500 Real-Time PCR System (Applied Biosystems). The Relative quantitation (RQ) of each mRNA was described using formula 2^(Ct interest gene - Ct endogenous control gene)^ and the sham vehicle group was used as the reference group to normalize the mRNA expression between the experimental groups [35].

### Determination of T Cell miRNA155 Expression

Enriched small RNA (10 ng) isolated from T cells was reverse transcribed to cDNA in a 15 µl reaction mixture using a miRNA155-specific RT primer and U6 snRNA-specific RT primer with TaqMan MicroRNA Reverse Transcription Kit according to the manufacturer's instructions (Applied Biosystems). The reaction was performed by one cycle at 16°C for 30 min, at 42°C for 30 min and the final cycle at 85°C for 5 min. The reaction was cooled to 4°C. 1.33 µl of RT product of miRNA155 and U6 snRNA were amplified with miRNA155 and U6 snRNA specific TaqMan MicroRNA Assays (Applied Biosystems). PCR reactions were performed at 95°C for 10 min, followed by 40 cycles at 95°C for 15 sec and 60°C for 1 min. U6 snRNA was used as an endogenous control for real-time quantitation of miRNA155 expression. The calculation of relative quantitation (RQ) of miRNA155 was performed as above for mRNA.

### Statistical Analysis

The data, wherever applicable, are presented as means ± standard error of the mean (SEM) and were analyzed with analysis of variance (ANOVA) with Tukey-Kramer Multiple Comparisons Test, or Student's two-tailed *t* test (In-Stat; GraphPad Software Inc., La Jolla, CA, USA). *P*<0.05 was considered statistically significant.

## Results

### Combined Ethanol and Burn Injury Decreases miRNA155 Expression in T Cells

**Figure 1:**
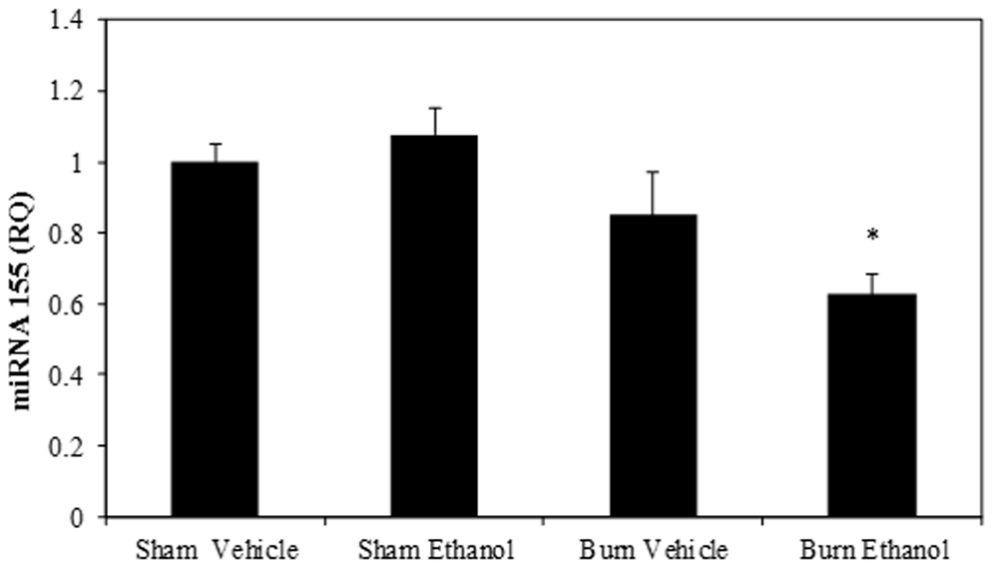
Ethanol intoxication combined with burn injury decreases miRNA155 expression in T cells. Splenic T cells (5×10^5^ cells/well) were cultured with plate-bound αCD3 (2 µg/ml) for 48 h and T cells were collected to determine miRNA155 expression by RT-PCR. U6 small nuclear RNA (U6 snRNA) was used as the endogenous control. Values are means ± SEM from four to twelve animals/per group. *p<0.01 compared to shams.

miRNAs are small non-coding RNAs which play a critical role in posttranscriptional modulation of T cell development and immune function. In this experiment, we determined whether acute ethanol intoxication combined with burn injury influences miRNA155 expression in T cells. To test this, T cells were cultured with plate-bound anti-CD3 (2 µg/ml) for 48 hours and the cells were harvested. Total miRNA was extracted and miRNA155 was detected using a specific TaqMan MicroRNA assay. U6 snRNA was used as an endogenous control for quantitation of miRNA155 expression. As shown in Fig. 1, there was no difference in T cell miRNA155 expression in sham mice regardless of ethanol intoxication. There was a tendency towards decreased miRNA155 expression in T cells isolated from the burn vehicle group of mice when compared to sham groups, but this was not found to be significant. However, there was a significant decrease in T cell miRNA155 expression in mice receiving ethanol intoxication combined with burn injury compared to shams (p<0.01).

Similar to miRNA155, we did not observe a significant difference in T cell IFN-γ in mice receiving ethanol or burn injury alone compared to sham vehicle one day after injury in our prior studies [12], [13]. Thus, the subsequent studies were carried out using sham vehicle and burn ethanol groups.

### Combined Ethanol and Burn Injury Decreases NFAT, Tbx21, Jun and Fox Expression in T Cells

**Figure 2:**
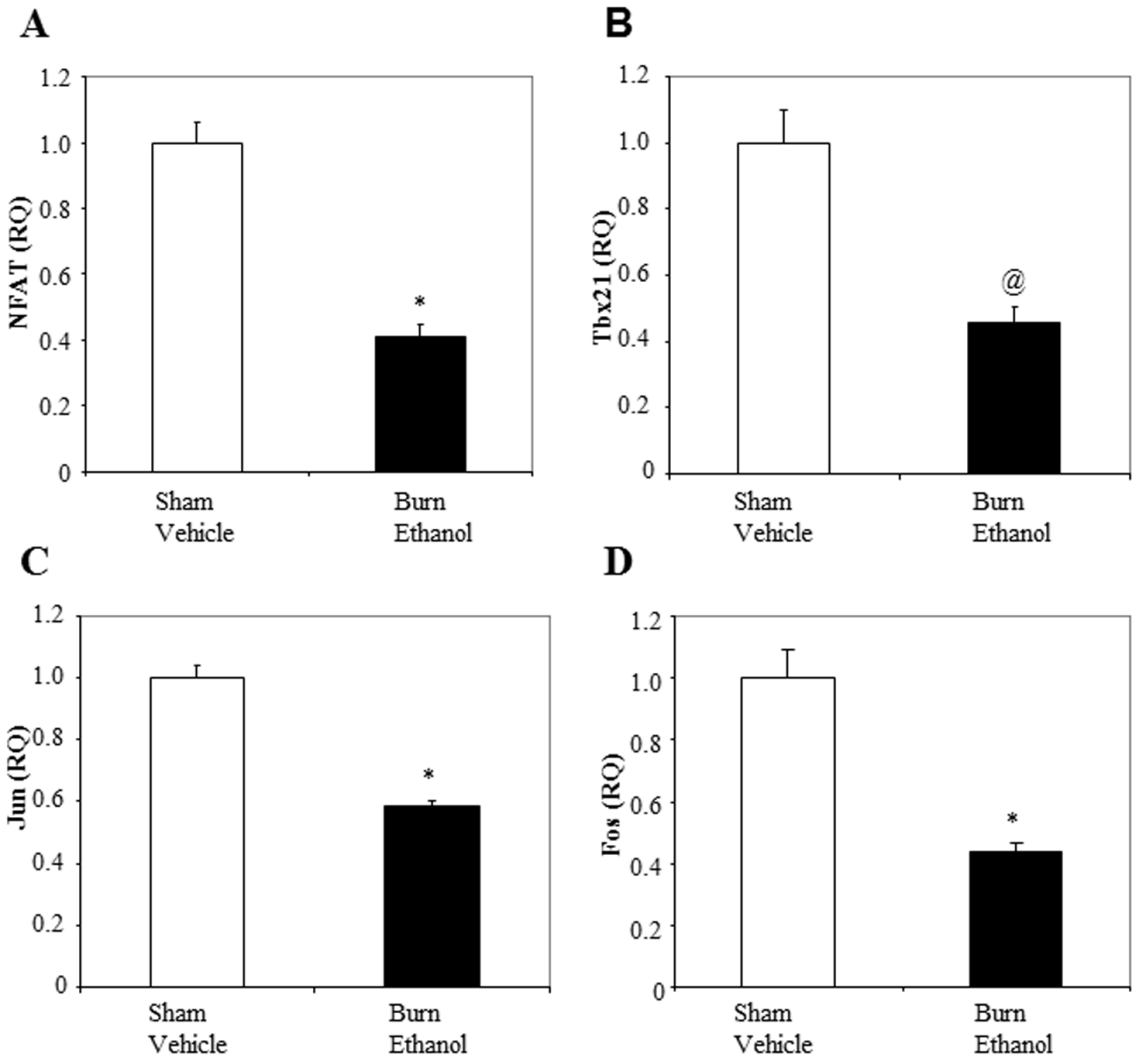
Ethanol intoxication combined with burn injury decreases NFAT, Tbx21, Jun and Fos expressions in T cells. Splenic T cells (5×10^5^ cells/well) were cultured with plate-bound αCD3 (2 µg/ml) for 48 h. T cells were collected to determine expression of NFAT (A), Tbx21 (B), Jun (C) and Fos (D) by RT-PCR. 18 s was used as the endogenous control. Values are means ± SEM from four animals/per group. *p<0.001 and @p<0.005 compared to sham vehicle.

Previous studies from our laboratory indicate that ethanol intoxication combined with burn injury results in decreased IFN-γ production, as well as suppressed p-38 and ERK1/2 phosphorylation in MLN and splenic T cells [10]–[13]. In the following experiment, we determined whether ethanol combined with burn injury also alters the expression of downstream transcription factors involved in T cell IFN-γ release. We measured expression of nuclear factors of activated T cells (NFAT), Tbx21 and AP-1 (Jun and Fox). As shown in Fig. 2, there was a significant decrease in the expression of NFAT (p<0.001), Tbx21 (p<0.005), Jun (p<0.001) and Fos (p<0.001) in T cells from mice receiving ethanol intoxication combined with burn injury compared to sham vehicle.

### Effects of rIL-12 and PMA/Ionomycin on T Cell IFN-γ Release Following Ethanol and Burn Injury

**Figure 3:**
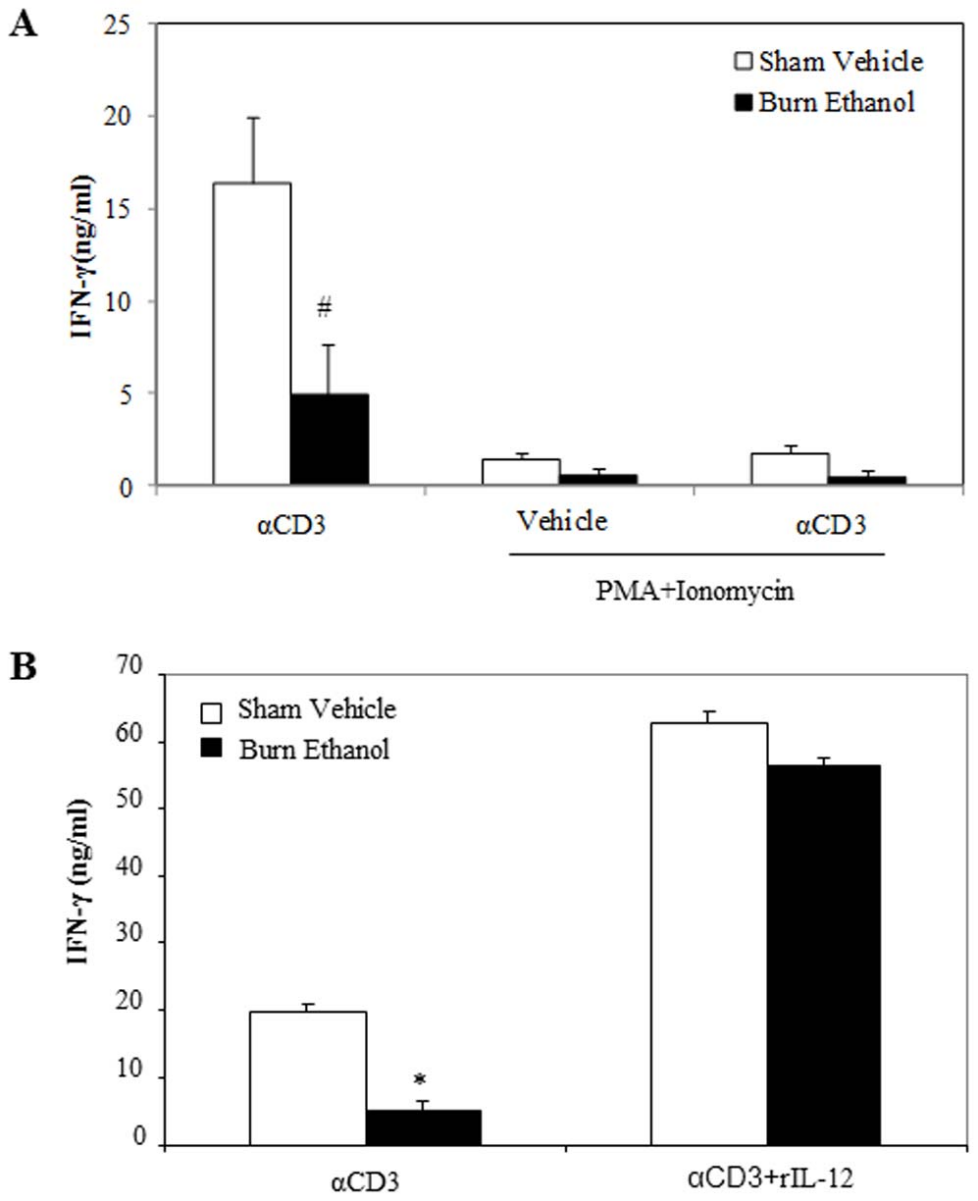
Effects of PMA/ionomycin and rIL-12 on T cell IFN-γ production following ethanol and burn injury. Splenic T cells (5×10^5^ cells/well) were cultured with plate-bound αCD3 (2 µg/ml) in the presence or the absence of PMA (10 ng/ml) plus ionomycin (50 ng/ml)(A) or rIL-12 (10 ng/ml) (B) for 48 h and supernatants were collected to determine IFN-γ production. Values are means ± SEM from six (A) to seven (B) animals/per group. *p<0.001 compared to other groups; #p<0.01 compared to respective sham vehicle.

We previously showed that treatment of T cells with rIL-12 normalized the release of IFN-γ by T cells following ethanol and burn injury [12]. Additionally, previous studies have utilized PMA and ionomycin to stimulate T cells by directly activating the protein kinase C (PKC) and calcium dependent signaling pathways [36]. In subsequent experiments, we utilized both rIL-12 and PMA/ionomycin to stimulate T cells to determine their effects on T cell IFN-γ release following ethanol and burn injury. In short, T cells were cultured with plate-bound anti-CD3 (2 µg/ml) in the presence or absence of PMA (10 ng/ml) plus ionomycin (50 ng/ml) or rIL-12 (10 ng/ml) for 48 h and supernatants were harvested to measure IFN-γ levels. These dosages of PMA, ionomycin and IL-12 were selected from our previously published studies [12], [14]. As shown in Fig. 3, there was a significant decrease in IFN-γ in T cells cultured with anti-CD3 alone in the burn ethanol group compared to sham vehicle group (p<0.01). T cells cultured with PMA plus ionomycin regardless of anti-CD3 stimulation further decreased IFN-γ production in the burn ethanol group, as well as in the sham vehicle group. The PMA plus ionomycin related decrease appears to be specific to IFN-γ. We also measured IL-2 production and cell viability after PMA plus ionomycin co-treatment. We found a several-fold increase in T cell IL-2 production and the cells were viable after PMA plus ionomycin co-treatment (data are not shown here). However, T cells cultured with anti-CD3 in the presence of rIL-12 significantly increased IFN-γ release compared to T cells cultured with anti-CD3 alone in T cells isolated from sham vehicle (3-fold higher) and burn ethanol (10-fold higher).

### Effects of rIL-12 and PMA/Ionomycin on T Cell Transcription Factor Expression Following Ethanol and Burn Injury

**Figure 4:**
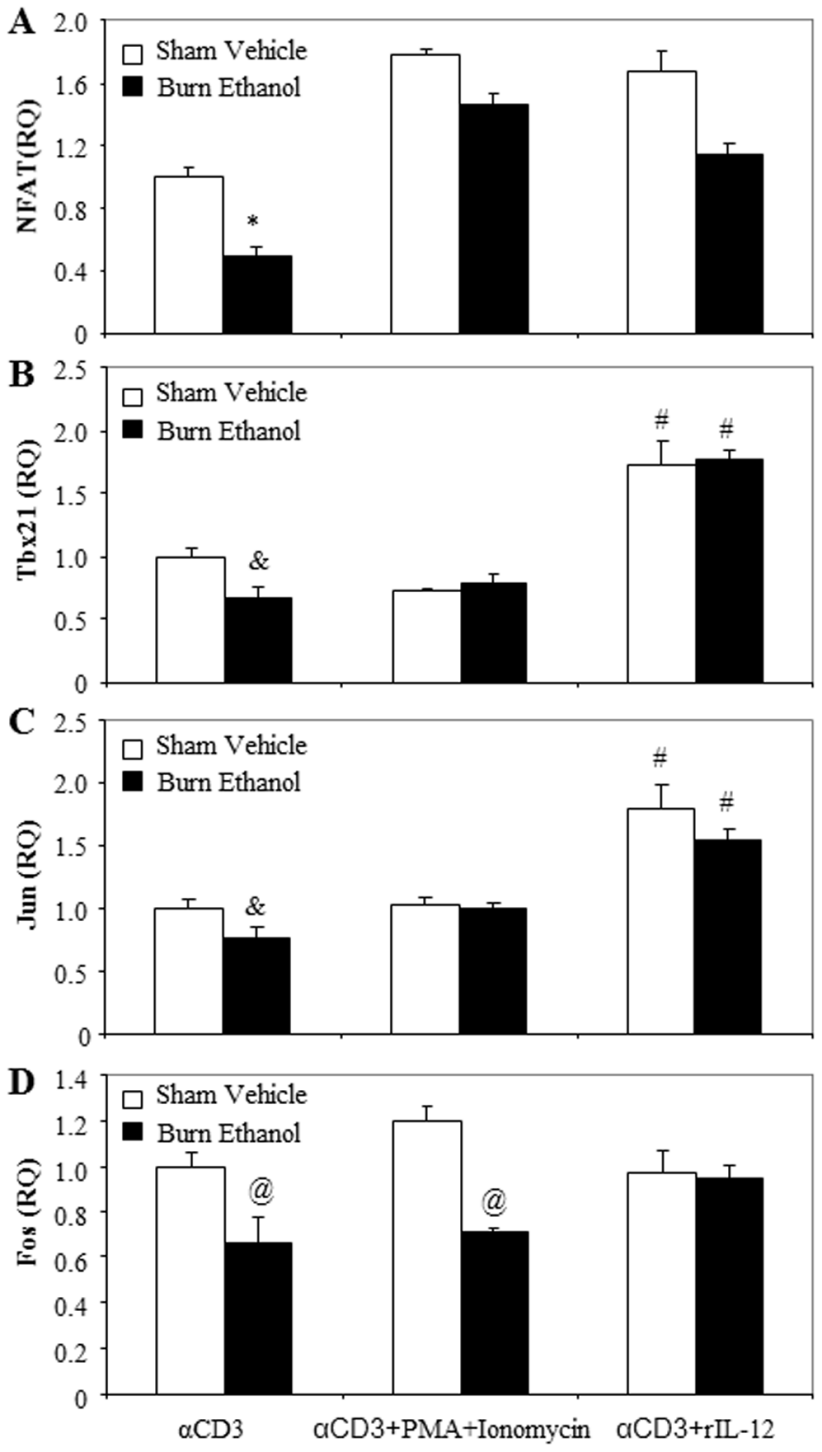
Effects of PMA/ionomycin and rIL-12 on T cell transcription factor expression following ethanol and burn injury. Splenic T cells (5×10^5^ cells/well) were cultured with plate-bound αCD3 (2 µg/ml) in the presence or absence of PMA (10 ng/ml) plus ionomycin (50 ng/ml), or rIL-12 (10 ng/ml) for 48 h. T cells were collected to determine NFAT (A), Tbx21 (B), Jun (C) and Fos (D) by RT-PCR. 18 s was used as the endogenous control. Values are means ± SEM from six animals/per group. *p<0.001 compared to other groups, #p<0.01 compared to respective αCD3, @p<0.05 compared to respective sham vehicle, &p<0.05 compared to respective sham vehicle.

We further examined the effect of rIL-12 and PMA plus ionomycin on T cell expression of NFAT, Tbx21, Jun and Fos. T cells were cultured with plate-bound anti-CD3 (2 µg/ml) in the presence or absence of PMA (10 ng/ml) plus ionomycin (50 ng/ml), or rIL-12 (10 ng/ml) for 48 h and cells were harvested to measure the expression of transcription factors. As shown in Fig. 4, there was a significant decrease in mRNA expression of NFAT, Tbx21, Jun and Fos in T cells cultured with anti-CD3 from burn ethanol mice compared to sham vehicle mice. The T cells cultured with anti-CD3 in the presence of PMA/ionomycin or rIL-12 prevented the decrease in NFAT expression (Fig. 4A) in burn ethanol mice. However, NFAT expression in the burn ethanol group did not reach the level of NFAT expression seen in sham vehicle (Fig. 4A). Treatment of T cells with rIL-12 also prevented suppression of Tbx21 (Fig. 4B, p<0.01), Jun (Fig. 4C, p<0.01) and Fos (Fig. 4D, p = 0.0512) expression in burn ethanol mice, however, the same was not seen in T cells treated with anti-CD3 in the presence of PMA/Ionomycin.

### Effects of rIL-12 and PMA/Ionomycin on T Cell miRNA155 Expression Following Ethanol and Burn Injury

**Figure 5:**
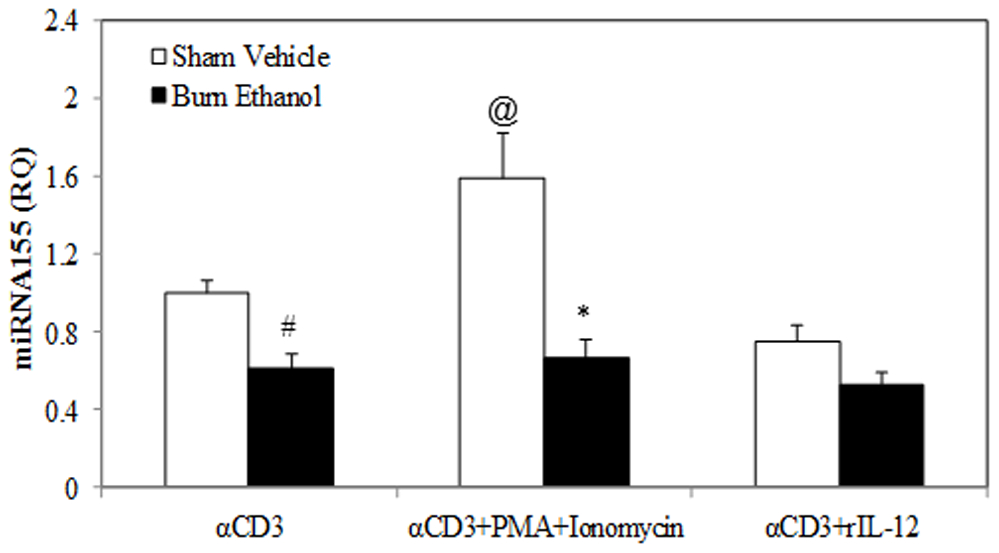
Effects of PMA/ionomycin and rIL-12 on T cell miRNA155 expression following ethanol and burn injury. Splenic T cells (5×10^5^ cells/well) were cultured with plate-bound αCD3 (2 µg/ml) in the presence or absence of PMA (10 ng/ml) plus ionomycin (50 ng/ml), or rIL-12 (10 ng/ml) for 48 h. T cells were collected to determine miRNA155 expression by RT-PCR. U6 snRNA was used as the endogenous control. Values are means ± SEM from six animals/per group. *p<0.001 compared to respective sham vehicle. #p<0.005 compared to respective sham vehicle. @p<0.05 compared to respective sham αCD3.

As shown in Fig. 5, we found a significant increase in miRNA155 expression in T cells from sham mice cultured with PMA/ionomycin in presence of anti-CD3. On the other hand, there was no difference in miRNA155 expression in T cells from sham mice cultured with anti-CD3 in the presence of rIL-12. Expression of miRNA155 in T cells from the burn ethanol group cultured with anti-CD3 in the presence of PMA/ionomycin or rIL-12 remained unaffected relative to corresponding sham vehicle groups.

### T Cell IFN-γ Release Is Independent of miRNA155

**Figure 6:**
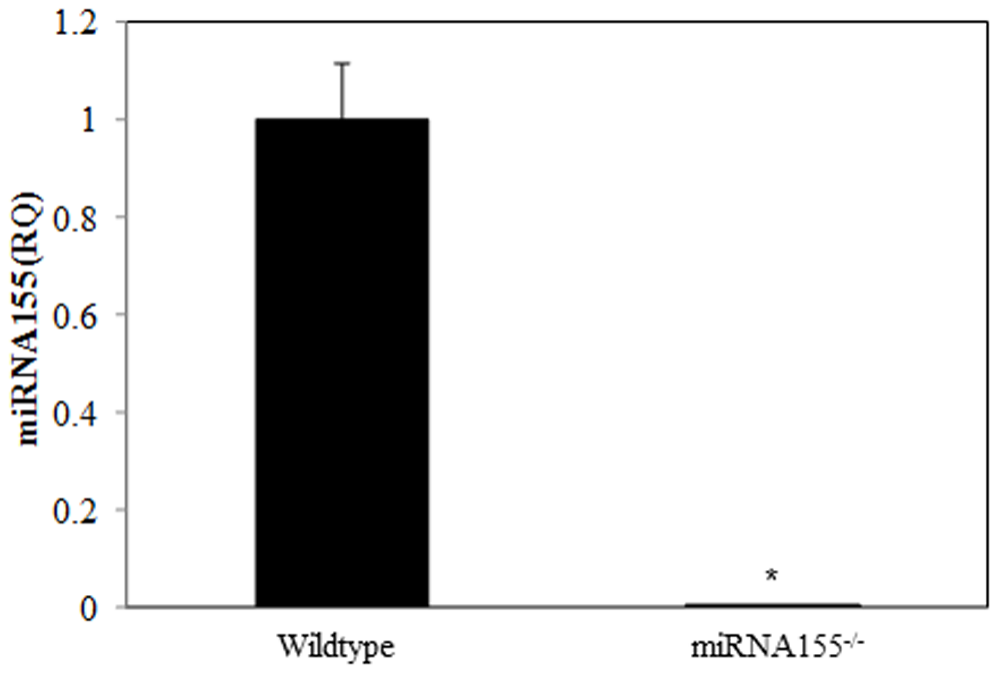
miRNA155 expression in T cells from wild-type mice and miRNA155^-/-^ mice. Splenic T cells (5×10^5^ cells/well) collected from wild-type or miRNA155^-/-^ mice were cultured with plate-bound αCD3 (2 µg/ml) for 48 h. T cells were collected to determine expression of miRNA155 by RT-PCR. U6 snRNA was used as the endogenous control. Values are means ± SEM from eight animals/per group. No expression of miRNA155 was observed in T cells from miRNA155-/- mice compared to wild-type mice. *p<0.001 compared to wildtype.

**Figure 7:**
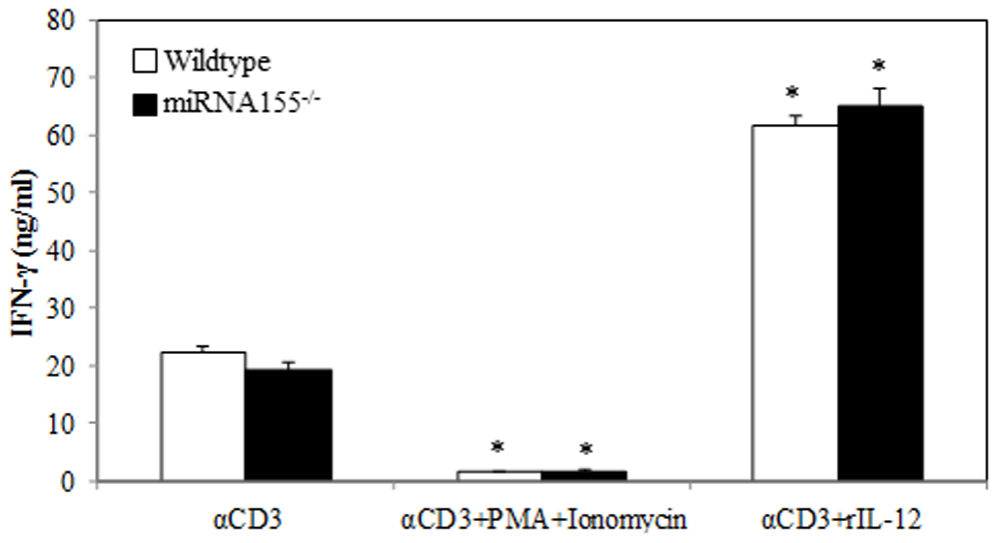
Effects of PMA/ionomycin and rIL-12 on miRNA155^-/-^ T cells IFN-γ production. Splenic T cells (5×10^5^ cells/well) collected from wild-type or miRNA155-/- mice were cultured with plate-bound αCD3 (2 µg/ml) in presence or absence of PMA (10 ng/ml) plus ionomycin (50 ng/ml) or rIL-12 (10 ng/ml) for 48 h and supernatants were collected to measure IFN-γ production. Values are means ± SEM from eight animals/per group. *p<0.05 compared to respective αCD3 groups.

Since miR155 has been shown to play a critical role in regulation of T cell IFN-γ release [25], [28], we used miRNA155^-/-^ mice in the following experiments to confirm whether miRNA155 is involved in T cell IFN-γ production. T cells were isolated from miRNA155^-/-^ and wild-type mice and were analyzed first for miRNA155 expression to confirm that T cells from miRNA155^-/-^ are indeed deficient in miRNA155. Results show no demonstrable miRNA155 expression in T cells isolated from miRNA155^-/-^ mice (Fig. 6). In subsequent experiments T cells were cultured with anti-CD3 in the presence or absence of PMA/ionomycin and rIL-12 for 48 h. Cell supernatants were collected to determine IFN-γ levels. As shown in Fig. 7, we observed that T cells cultured with anti-CD3 in the presence of PMA/ionomycin produced significantly lower IFN-γ when compared to T cells cultured with anti-CD3 alone. In contrast, T cells cultured with anti-CD3 in the presence of rIL-12 produced significantly more IFN-γ as compared to T cells cultured with anti-CD3 alone. However, there was no difference in IFN-γ production between miRNA155^-/-^ and wild-type mice within each treatment group.

### miRNA155 Does Not Influence Transcription Factors Expression

**Figure 8:**
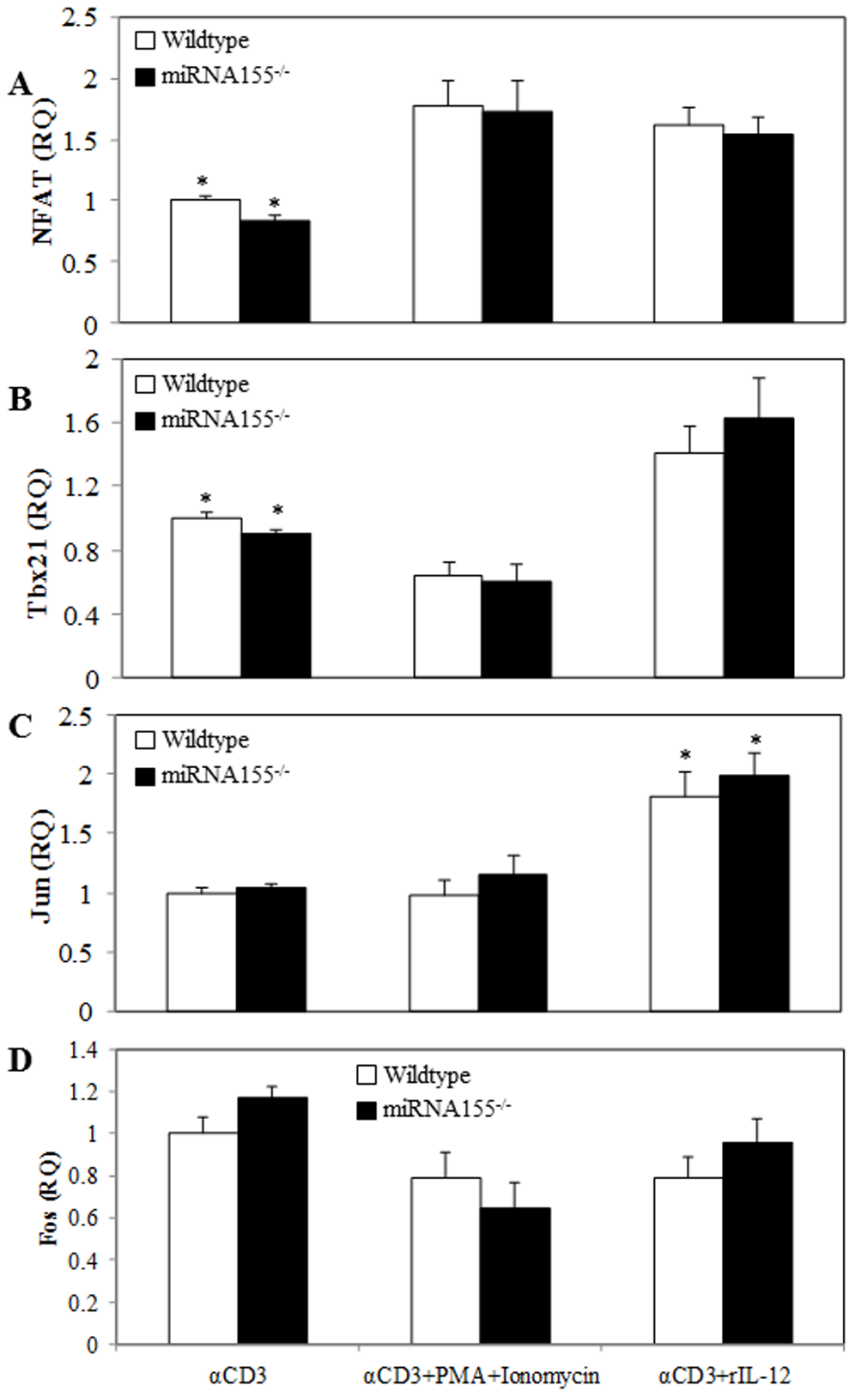
Effects of PMA/ionomycin and rIL-12 on miRNA155^-/-^ mice T cell transcription factor expression. Splenic T cells (5×10^5^ cells/well) collected from wild-type or miRNA155^-/-^ mice were cultured with plate-bound αCD3 (2 µg/ml) in the presence or the absence of PMA (10 ng/ml) plus ionomycin (50 ng/ml), or rIL-12 (10 ng/ml) for 48 h. T cells were collected to determine NFAT, Tbx21, Jun and Fos expression by RT-PCR. GAPDH was used as the endogenous control. Values are means ± SEM from eight animals/per group. *p<0.05 compared to other respective groups.

As shown in Fig. 8, we noted a significant increase in NFAT, Tbx21 and Jun, but not Fos expression in T cells cultured with anti-CD3 plus rIL-12 compared to T cells stimulated with anti-CD3 alone in both miRNA155^-/-^ and wild-type mice. There was a significant increase in NFAT and a decrease in Tbx21 expression in T cells cultured with anti-CD3 plus PMA/ionomycin compared to T cells stimulated with anti-CD3 alone in both strains of mice. However, there were no differences in T cell expression of NFAT, Tbx21, Jun or Fos between miRNA155^-/-^ and wild-type mice within each treatment group.

## Discussion

Multiple studies have demonstrated decreased T cell IL-2 and IFN-γ production following burn injury [19]–[21], [37]–[39]. We found that ethanol intoxication at the time of burn injury further potentiates the suppression in T cell IL-2 and IFN-γ production after burn injury [10]–[13]. In this study, we examined whether this is due to derailments in expression of T cell miRNA155 and/or downstream transcription factors NFAT, Tbx21 and AP-1 (Jun and Fos). We found a significant decrease in T cell NFAT, Tbx21, Jun and Fos expression following ethanol and burn injury. This was accompanied with a significant decrease in T cell miRNA155 expression in mice receiving ethanol combined with burn injury compared to those receiving sham injury. Recent studies indicate that miRNAs play essential roles in the regulation of T cell effector functions [25], [40], [41]. miRNAs are transcribed by RNA polymerase II in the nucleus, and are cleaved into precursor miRNA (pre-miRNA) by a complex containing Drosha, an RNAase enzyme, and DGCR8, a double-stranded-RNA-binding protein. These pre-miRNAs are then exported to the cytoplasm and further processed by Dicer, an RNAase-III family member, to produce 20–25 nucleotide long single-stranded mature miRNAs. Mature miRNAs are loaded onto the RNA-induced silencing complex (RISC) to target mRNA 3′untranslated regions (UTRs), resulting in either post-transcriptional repression or degradation of target mRNAs [24], [40], [42].

A single miRNA may target multiple genes, similarly a single gene can be regulated by more than one miRNA. Hundreds of miRNAs are present in lymphocytes (B and T cells), macrophages and dendritic cells, and are involved in their development and function [24], [40], [42]. miRNA155 is specifically reported to be involved in protective immunity. Mice deficient in bic/microRNA-155(bic^m2/m2^) exhibit defective adaptive immunity. Bic^m2/m2^ immunized with enteric pathogen *Salmonella typhimurium (aroA* mutant strain) were not protected against this bacterium and demonstrated enhanced mortality after challenge. Similarly, T cells from bic^m2/m2^ mice immunized with the T cell-dependent antigen, tetanus toxin fragment C protein (TetC), failed to produce IL-2 or IFN-γ. Furthermore, dendritic cells from Bic^m2/m2^ mice failed to efficiently activate T cells [25]. Moreover, mice deficient in miRNA155 are biased toward Th2 cell differentiation and display enhanced levels of Th2 cytokines IL-2, IL-5 and IL-10 [23], [25], [43]. miRNAs can influence intracellular signaling pathways and thus regulate multiple biological processes and functions. miRNA155 directly targets SHIP1 by 3′URT interactions and directly suppresses expression of homology-2 domain-containing inositol 5-phosphatase 1 (SHIP1), which induces dephosphorylation of PIP3 and PIP2 and downregulates the PI3K pathway [44]. SHIP-deficient T cells are skewed towards a Th1 phenotype and have diminished Th2 cytokine production following an immune challenge [45]. Human miRNA155 overexpressing NK cells stimulated with IL-12 plus IL-18 produce significantly more IFN-γ by down-regulating SHIP 1 [46]. B cell receptor (BCR) activation requires BIC/miRNA155 by ERK and JNK signaling pathway. B cells treated with inhibitors of ERK or JNK suppress BIC/miRNA expression, as well as inhibit AP-1 family members, Jun B, c-Fos and Fos B, but not c-Jun. The mutation of the AP-1 site decreased BIC promoter activity and altered BCR response [47]. NFkB also has a putative binding site in the miRNA155 promoter [48]. Taken together, these data suggest that miRNA155 plays a key role in the immune homeostasis and in protection against infection.

Previously, we have shown that ethanol intoxication combined with burn injury suppresses p38 and ERK1/2 phosphorylation in T cells [10]–[13]. Inhibition of T cell MAPKs following ethanol and burn injury could result from a decrease in upstream signaling molecules as observed in the activation of P59^fyn^ and other protein tyrosine kinases associated with TCR [11], [49], [50]. Such decreases in these upstream molecules may cause a decrease in Ca^2+^ and PKC. Indeed, the activation of both of these molecules is implicated in T cell activation and proliferation [51]. In this study, we used PMA/ionomycin to bypass the TCR and directly induce Ca^2+^ and PKC-dependent pathways in T cells [14], [36]. Studies have shown that activation of both Ca^2+^ signaling and PKC contribute to the activation of T cell nuclear factors such as NFAT and AP-1 [52]–[54]. We found that treatment of cells with PMA/ionomycin did not prevent the decrease in expression of miRNA155, nuclear factors and IFN-γ after ethanol and burn injury. These findings suggests that stimulation of T cells with PMA and ionomycin may not be sufficient to induce the signaling mechanism needed for IFN-γ release.

We then utilized IL-12, a cytokine which is involved in Th1 differentiation and release of IFN-γ [55], [56]. Previously, we have demonstrated that the ex vivo treatment of T cells with IL-12 prevents the decrease in IFN-γ [12]. IL-12 induces the transcription factor T-bet (Tbx21 in mice) in naïve T cells and promotes their differentiation towards a Th1 subset. Since miRNA155 is involved in Th1 differentiation and IFN-γ release, we treated T cells with rIL-12 and examined the effects of IL-12 on miRNA155 and nuclear factor expression following ethanol combined with burn injury. We observed that T cells treated with anti-CD3 plus rIL-12 restored NFAT, Tbx21, Jun and Fos, as well as the release of IFN-γ. However, it did not influence the expression of miRNA155 in T cells. T cells cultured with rIL-12 continue to exhibit a similar decrease in miRNA155 following ethanol and burn injury. To further delineate the role of miR155, T cells from miRNA155^-/-^ mice were examined for their ability to release IFN-γ and expression of nuclear factors. We found that T cells cultured with anti-CD3 plus PMA/ionomycin significantly decreased in IFN-γ production compared to T cells cultured with anti-CD3 alone. In contrast, T cells cultured with anti-CD3 plus rIL-12 significantly enhanced IFN-γ production compared to T cells cultured with anti-CD3. However, there was no difference in T cell IFN-γ production between miRNA155^-/-^ and wild-type mice. Consistent with this observation, there were no differences in NFAT, Tbx21 and Jun and Fos in T cells isolated from miRNA155^-/-^ compared to wild-type mice regardless of treatment. Together, our findings suggest that while the ethanol and burn injury decreases the expression of miRNA155, it may not be involved in decreased IFN-γ following ethanol and burn injury. Moreover, IL-12 restoration of T cell IFN-γ and transcription factors is independent of miRNA155. In contrast, Oertli M. et al reported that CD4^+^CD25^-^T cells from miRNA155^-/-^ mice stimulated with CD3/CD28 in presence of 10 ng/ml rIL-2 reduced proliferation and IFN-γ production, as well as IL-17 production compared to wildtype T cells [57]. While the reasons for such conflicting results remain unknown, it is likely that in the absence of miRNA155, other compensatory mechanisms are activated in IL-12 restoration of IFN-γ after ethanol and burn injury. Similarly, T cells from bic/miRNA-155^-/-^ mice cultured under Th1 conditions (IL-12+anti-IL-4) produced same amount of IFN-γ as wild-type T cells. Bic/miRNA155^-/-^ T cells cultured under Th2 conditions (IL-4+anti-IFN-γ+anti-IL-12) also produced same amount of IL-4 as wild-type T cells. However, T cells cultured with limited IL-4 (12.5 U/ml) promoted differentiation of T cells into Th2 cells. Bic/miRNA155^-/-^ T cells produced more IL-4 and less IFN-γ than wild-type T cells [23]. In summary, these findings suggest that T cell activation and differentiation is complex and involves a multitude of factors ranging from the external cytokine milieu to the activation of signaling pathways. Thus more studies are needed to understand this complexity. While miRNA155 is not involved in T cell suppression after alcohol and burn injury, additional studies should be carried out to explore the role of other miRNAs (e.g. miRNA126, miRNA181a and miRNA182), implicated in T cell development and differentiation, in suppressed T cell IFN-γ after alcohol and burn injury.
